# Airflow Obstruction and Cardio-metabolic Comorbidities

**DOI:** 10.1080/15412555.2019.1614550

**Published:** 2019-05-27

**Authors:** Filip J.J. Triest, Michael Studnicka, Frits M.E. Franssen, William M. Vollmer, Bernd Lamprecht, Emiel F.M. Wouters, Peter G. J. Burney, Lowie E.G.W. Vanfleteren

**Affiliations:** a CIRO+, Centre of expertise for chronic organ failure, Horn, the Netherlands;; b Department of Respiratory Medicine, MUMC+, Maastricht University Medical Centre, Maastricht, the Netherlands;; c Department of Respiratory Medicine, AZ Sint-Lucas, Gent, Belgium;; d Department of Pneumology, Paracelsus Medical University, Salzburg, Austria;; e Kaiser Permanente Center for Health Research, Portland, OR, USA;; f Department of Pulmonary Medicine, Kepler-University-Hospital, Linz, Austria;; g Faculty of Medicine, Johannes-Kepler-University, Linz, Austria;; h Respiratory Epidemiology, Occupational Medicine and Public Health, National Heart and Lung Institute, Imperial College, London, UK;; i COPD Center, Sahlgrenska University Hospital, Institute of Medicine, Gothenburg University, Gothenburg, Sweden

**Keywords:** Airflow obstruction, COPD, comorbidity, cardiovascular, hypertension, diabetes

## Abstract

Chronic obstructive pulmonary disease (COPD) is characterized by airflow obstruction and often co-exists with cardiovascular disease (CVD), hypertension and diabetes. This international study assessed the association between airflow obstruction and these comorbidities. 23,623 participants (47.5% males, 19.0% current smokers, age: 55.1 ± 10.8 years) in 33 centers in the Burden of Obstructive Lung Disease (BOLD) initiative were included. 10.4% of subjects had airflow obstruction. Self-reports of physician-diagnosed CVD (heart disease or stroke), hypertension and diabetes were regressed against airflow obstruction (post-bronchodilator FEV_1_/FVC < 5th percentile of reference values), adjusting for age, sex, smoking (including pack-years), body mass index and education. Analyses were undertaken within center and meta-analyzed across centers checking heterogeneity using the I^2^-statistic. Crude odds ratios for the association with airflow obstruction were 1.42 (95% CI: 1.20–1.69) for CVD, 1.24 (1.02–1.51) for hypertension, and 0.93 (0.76–1.15) for diabetes. After adjustment these were 1.00 (0.86–1.16) (I^2^:6%) for CVD, 1.14 (0.99–1.31) (I^2^:53%) for hypertension, and 0.76 (0.64–0.89) (I^2^:1%) for diabetes with similar results for men and women, smokers and nonsmokers, in richer and poorer centers. Alternatively defining airflow obstruction by FEV_1_/FVC < 2.5th percentile or 0.70, did not yield significant other results. In conclusion, the associations of CVD and hypertension with airflow obstruction in the general population are largely explained by age and smoking habits. The adjusted risk for diabetes is lower in subjects with airflow obstruction. These findings emphasize the role of common risk factors in explaining the coexistence of cardio-metabolic comorbidities and COPD.

## Introduction

Non-communicable diseases, including cardiovascular disease (CVD), cancer, diabetes and chronic respiratory disease are responsible for almost 70% of global deaths, the majority occurring in low- and middle-income countries (1). Chronic obstructive pulmonary disease (COPD) is characterized by persistent respiratory symptoms and airflow obstruction that is due to airway and/or alveolar abnormalities (2). COPD and CVD often co-exist (3). A meta-analysis by Chen et al. reported a nearly 2.5 times increased risk of CVD in patients with COPD (4). Hypertension, diabetes and smoking, which all increase the risk for CVD, were also more often reported in COPD (4). All the studies included in that meta-analysis were conducted in Western countries and most of them defined COPD by physician diagnosis and not by spirometry. As COPD is often misdiagnosed (5), these analyses might not provide an accurate account of the relationship between airflow obstruction and comorbidities.

Within the Burden of Obstructive Lung Disease (BOLD) study, an international population-based study in 29 countries with different ethnic and cultural backgrounds, we have analyzed the association between the presence of spirometrically identified airflow obstruction and the prevalence of self-reported physician-diagnosed CVD, hypertension and diabetes, taking into account other risk factors.

## Methods

### Study Design and Participants

The design and rationale for the BOLD initiative have been previously published (6). Sampling plans were used for the recruitment of representative non-institutionalized participants for all study sites. As of November 2016, 33 sites (Table 1) had completed data collection and were included in this analysis. Each participating site aimed to recruit a sample of at least 300 men and 300 women who were not institutionalized, aged ≥40 years, and living in a well-defined administrative area in which the total population exceeded 150,000. Participants were interviewed by means of a questionnaire and underwent a standardized assessment. Approval was obtained from each local ethics committee, and written informed consent was obtained from each participant. Subjects, aged 40–89 years, with a usable post-bronchodilator lung function and a valid smoking history were extracted from the BOLD database. We then selected those with full data on subject characteristics (age, sex, smoking status, body mass index (BMI) and education) and comorbidities of CVD, hypertension, and diabetes.

**Table 1.** General characteristics of study participants at each site and overall.

Site, Country	*N*	Smoking Status (%)			Sex (%)	Age (years)		BMI (kg/m^2^)		AO (%)	Comorbidities (%)			GNI
		Current	Ex	Never	Male	Mean ± SD		Mean ± SD			CVD	H	D	High
Adana, Turkey	806	34.9	19.9	45.3	48.3	53.6 ± 10.4		29.6 ± 5.3		14.3	11.8	27.0	10.3	no
Annaba, Algeria	862	16.7	21.9	61.4	49.8	52.9 ± 9.7		28.3 ± 5.7		7.0	6.6	22.2	14.4	no
Bergen, Norway	656	26.2	36.6	37.2	49.2	59.7 ± 12.5		26.5 ± 4.3		12.5	15.4	29.4	5.9	yes
Blantyre, Malawi	399	3.8	9.3	87.0	39.8	52.2 ± 9.7		25.1 ± 5.4		8.3	2.5	20.1	6.0	no
Cape Town, SA	833	46.3	21.4	32.3	37.0	54.0 ± 10.2		27.9 ± 7.4		18.6	13.3	38.9	13.2	no
Chui, Kyrgyztan	858	19.8	9.7	70.5	31.5	53.0 ± 8.8		28.5 ± 5.6		10.0	16.6	29.7	5.7	no
Colombo, Srilanka	1020	12.9	7.5	79.6	44.6	53.7 ± 9.4		24.2 ± 4.6		7.3	5.9	20.6	13.4	no
Cotonou, Benin	677	1.8	0.1	98.1	43.9	51.5 ± 9.3		26.4 ± 5.5		7.7	5.3	29.8	2.5	no
Fes, Morocco	758	8.6	18.7	72.7	46.0	55.2 ± 10.0		27.9 ± 5.3		9.6	5.8	33.1	14.6	no
Guangzhou, China	471	29.9	14.0	56.1	49.9	54.0 ± 10.6		23.3 ± 3.3		7.9	9.8	17.6	4.0	no
Hannover, Germany	681	20.7	39.4	39.9	51.2	58.0 ± 10.9		27.3 ± 4.6		8.8	17.0	38.3	6.3	yes
Ife, Nigeria	859	2.6	7.9	89.5	39.1	55.5 ± 11.5		25.4 ± 5.4		6.9	0.2	2.3	0.8	no
Krakow, Poland	522	29.3	32.4	38.3	50.8	55.6 ± 11.4		27.7 ± 4.7		13.6	32.4	42.0	11.1	yes
Lexington, USA	505	26.5	33.9	39.6	40.4	56.5 ± 9.8		30.8 ± 6.8		15.0	29.3	49.1	17.4	yes
Lisbon, Portugal	709	13.3	26.8	59.9	46.7	63.3 ± 11.3		28.2 ± 4.7		11.6	17.5	37.5	11.0	yes
London, England	672	21.0	41.2	37.8	48.1	58.0 ± 11.4		27.1 ± 5.0		15.8	7.1	33.0	6.5	yes
Maastricht, NL	589	22.9	42.4	34.6	50.8	57.5 ± 10.6		27.4 ± 4.5		18.2	17.0	29.5	7.3	yes
Manila, Philippines	890	32.7	20.2	47.1	42.2	52.2 ± 10.1		24.9 ± 4.7		8.5	11.0	26.5	6.0	no
Mumbai, India	440	6.6	3.2	90.2	62.5	51.1 ± 8.9		23.8 ± 4.0		6.8	2.3	10.0	5.2	no
NT, Philippines	722	35.9	16.8	47.4	49.3	54.1 ± 10.5		21.5 ± 3.9		14.3	8.3	19.7	2.6	no
Naryn, Kyrgyztan	816	15.1	9.8	75.1	38.5	53.2 ± 9.7		27.0 ± 5.0		7.4	11.6	15.7	1.0	no
Penang, Malaysia	646	20.3	5.0	74.8	50.9	54.8 ± 9.3		26.0 ± 4.5		4.0	2.8	25.2	14.4	no
Pune, India	843	8.9	3.0	88.1	59.4	52.4 ± 9.8		22.1 ± 3.8		6.2	1.4	5.1	2.1	no
Reykjavik, Iceland	755	18.4	42.5	39.1	53.1	56.3 ± 11.6		27.9 ± 4.9		11.0	15.4	32.1	4.8	yes
Riyadh, Saudi Arabia	654	7.8	17.0	75.2	54.9	50.5 ± 7.5		31.2 ± 6.0		3.1	6.7	26.6	29.4	no
Salzburg, Austria	1255	19.3	33.5	47.2	54.3	57.6 ± 11.3		26.4 ± 4.2		15.8	12.5	28.9	6.4	yes
Sousse, Tunisia	658	26.7	13.2	60.0	47.0	53.0 ± 9.0		29.3 ± 5.6		5.0	5.6	21.0	10.9	no
Srinagar, India	739	10.3	1.9	87.8	54.9	51.7 ± 10.3		22.4 ± 3.6		16.4	1.4	27.1	2.2	no
Sydney, Australia	423	14.9	36.6	48.5	49.6	58.5 ± 11.9		28.0 ± 5.1		10.2	12.8	29.8	8.5	yes
Tartu, Estonia	611	18.2	29.3	52.5	50.2	60.8 ± 12.0		28.4 ± 5.2		7.0	37.3	40.1	7.2	yes
Tirana, Albania	926	21.8	15.2	63.0	49.8	54.7 ± 10.6		28.1 ± 4.7		8.6	4.2	22.8	6.5	no
Uppsala, Sweden	547	14.3	43.1	42.6	51.7	58.4 ± 10.9		27.0 ± 4.4		9.3	11.0	28.7	3.8	yes
Vancouver, Canada	821	13.9	38.4	47.7	41.7	55.8 ± 11.5		26.7 ± 5.2		12.3	12.8	20.2	7.1	yes
**Total: n = 23623**		**19.0**	**21.1**	**59.8**	**47.5**	**55.1 ± 10.8**		**26.7 ± 5.5**		**10.4**	**10.8**	**26.2**	**8.1**	**37%**

Abbreviations: BMI: body mass index. AO: airflow obstruction. GNI: gross national income. CVD: cardiovascular disease. H: hypertension. D: diabetes. SA: South Africa. USA: United States of America. NL: The Netherlands. NT: Nampicuan&Talugtug

### Assessments

#### Questionnaire

Questionnaire data were obtained by face-to-face interviews conducted by trained, certified staff in the participant's native language. All participants completed a core questionnaire, based on standardized instruments, which included information on risk factors for airflow obstruction and comorbidities (6). We asked a standardized dichotomous question for self-reported physician-diagnosed comorbidities heart disease, stroke, hypertension or diabetes. For example: “Has a doctor or other health care provider ever told you that you had *heart disease*?”

#### Anthropometry

Body height was measured to the nearest 0.5 cm. Body weight was assessed to the nearest 0.1 kg after emptying the bladder, with the subjects standing barefoot and wearing light indoor clothing. Body mass index was calculated as body weight/height^2^ (kg/m^2^).

#### Spirometry

Lung function data were collected using the ndd EasyOne Spirometer (ndd Medical Technologies, Zurich, Switzerland) (7). Lung function was measured 15 minutes after administration of 200 μg of salbutamol with a metered dose inhaler with Volumatic spacer (GlaxoSmithKline, Uxbridge, England). Local spirometry technicians were trained and certified. All spirograms were reviewed centrally based on standardized criteria and were deemed usable if they fully met ATS acceptability criteria and were reproducible to within 200 mL (8).

### Definitions

Airflow obstruction was defined according to the lower limit of normal (LLN, <5th percentile) of the forced expiratory volume in the first second/forced vital capacity (FEV_1_/FVC) ratio calculated from the normal values for white Americans by the National Health and Nutrition Examination Survey (NHANES) III (9). Alternative definitions for airflow obstruction were used in additional exploratory analyses: (a) FEV_1_/FVC < 2.5th percentile to select a more symptomatic population. A lower FEV_1_/FVC ratio is a predictor of increased dyspnea and declined quality of life, mental and physical health (10); (b) the fixed FEV_1_/FVC ratio < 0.70 to define airflow obstruction, as proposed by the Global Initiative for Chronic Obstructive Lung Disease (GOLD) (2), to evaluate the effect of misclassification of airflow obstruction by using the fixed ratio (11). A current or ex-smoker was defined as a person who had smoked >20 packs of cigarettes in a lifetime or >1 cigarette/day for a year. An ex-smoker was further defined by a self-report of having stopped smoking (no period restriction). CVD was defined as the presence of either heart disease or stroke. Sites were categorized as high gross national income if they were located in countries with high-income economies as defined by the World Bank (gross national income per capita ≥$12,745, using the Atlas method) (12).

### Statistical analysis

Statistics were performed in STATA, version 13.1 (Stata Corporation, College Station, TX, USA). For each site, we used logistic regression to analyze the association of diabetes, hypertension, and CVD with airflow obstruction. Models were fitted with and without adjustment for known cardiovascular risk factors: sex (13), education (14) (less than high school, high school, college), BMI (15) (≤18, 18–25, 25–30, >30 kg/m^2^), age (13) (40–49, 50–59, 60–69, 70–89 years), smoking status (16) (never, former, current), and pack-years (17) (never, 0–10, 10–20, 20–30, >30). All regression models, both adjusted and unadjusted, accounted for clustering within each site.

Random effects meta-analyses were performed using, for each site, the odds ratios of diabetes, hypertension, and CVD in subjects with airflow obstruction compared to those without airflow obstruction. To better appreciate the relative influence of the covariates in our model, we fitted (1.) the full model with all covariates, (2.) the same model but leaving out each covariate one by one (i.e., six models each including five of the six variables from the full model), and (3.) a model without any adjusting variables. Additional meta-analyses were performed stratified by, respectively, sex, smoking history (never vs. ever-smoker), and economy (high vs. low- & middle-income economies). Study sites that reported <20 participants with a specific comorbidity or with singularity in the data (i.e. no one with both COPD and that specific comorbidity) were excluded from the meta-analysis because these sites should not be fitted in the model. These sites are noted in the legends of the meta-analyses. *P*-values < 0.05 were considered statistically significant. Statistical heterogeneity was assessed by *I*^2^ and Chi-square testing. The ln(odds ratios) of the stratified meta-analyses were compared using *Z*-scores.

## Results

### Study population characteristics

**Figure 1.**
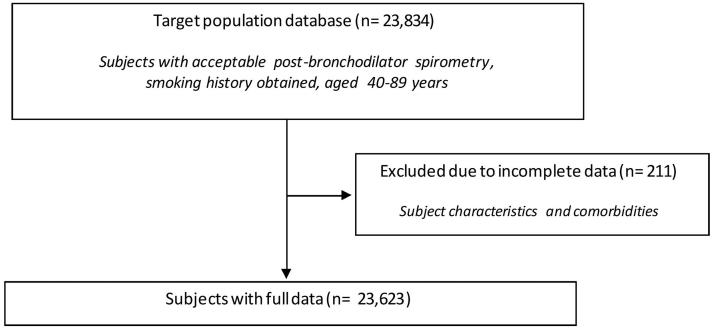
Flow chart of data extraction.

23,623 subjects had full data with regard to post-bronchodilator spirometry, covariates, and comorbidities (Figure 1). Less than 1% (*n* = 211) of subjects were excluded from further analysis. Characteristics of excluded subjects are shown in Supplementary Table 1. The majority of all subjects were never smokers, and one in five were current smokers (Table 1). Current and former smoking was more than twice as prevalent in males compared to females (data not shown). Thirteen out of 33 study sites were located in countries with a high-income economy. Overall, participants had a mean age of 55.1 ± 10.8 years, were slightly overweight, and approximately half of them were males. As defined by the LLN in this study (FEV_1_/FVC < 5th percentile), 10.4% of the study population had airflow obstruction, while 8.0% of the study population had a FEV_1_/FVC < 2.5th percentile. According to the fixed ratio (FEV_1_/FVC < 0.70) definition, 15.1% of the study population would have airflow obstruction. Of these, 33.7% did not have airflow obstruction, as defined by the LLN, while 3.6% vice versa. The highest prevalence of airflow obstruction (LLN) was noted in Cape Town (South Africa) (18.6%), Maastricht (Netherlands) (18.2%) and Srinagar (India) (16.4%).

### Prevalence of comorbidities

The prevalence of CVD (heart disease or stroke), hypertension and diabetes was respectively 10.8%, 26.2% and 8.1% overall (Table 1), 10.9%, 28.9%, 8.2% for females, and 10.7%, 23.2%, 7.8% for males (data not shown). The highest prevalences of CVD were noted in Tartu (Estonia) (37.3%), Krakow (Poland) (32.4%) and Lexington (USA) (29.3%), whereas the lowest prevalences were reported in Ife (Nigeria) (0.2%), Pune (India) (1.4%) and Srinagar (India) (1.4%).

### The unadjusted association between airflow obstruction and comorbidities

In meta-analyses of the unadjusted odds ratios, the presence of airflow obstruction (LLN) was significantly associated with increased risk of CVD (crude OR 1.42, 95% CI 1.20–1.69) and hypertension (crude OR 1.24, 95% CI 1.02–1.51), but not diabetes (crude OR 0.93, 95% CI 0.76–1.15). Similar results were found using the alternative definitions for airflow obstruction, although the increased risk of hypertension was no longer significant using the FEV_1_/FVC < 2.5th percentile definition and the risk of all three comorbidities tends to increase using the fixed ratio (FEV_1_/FVC < 0.70) (Figure 5).

### The adjusted association between airflow obstruction and comorbidities

**Figure 2.**
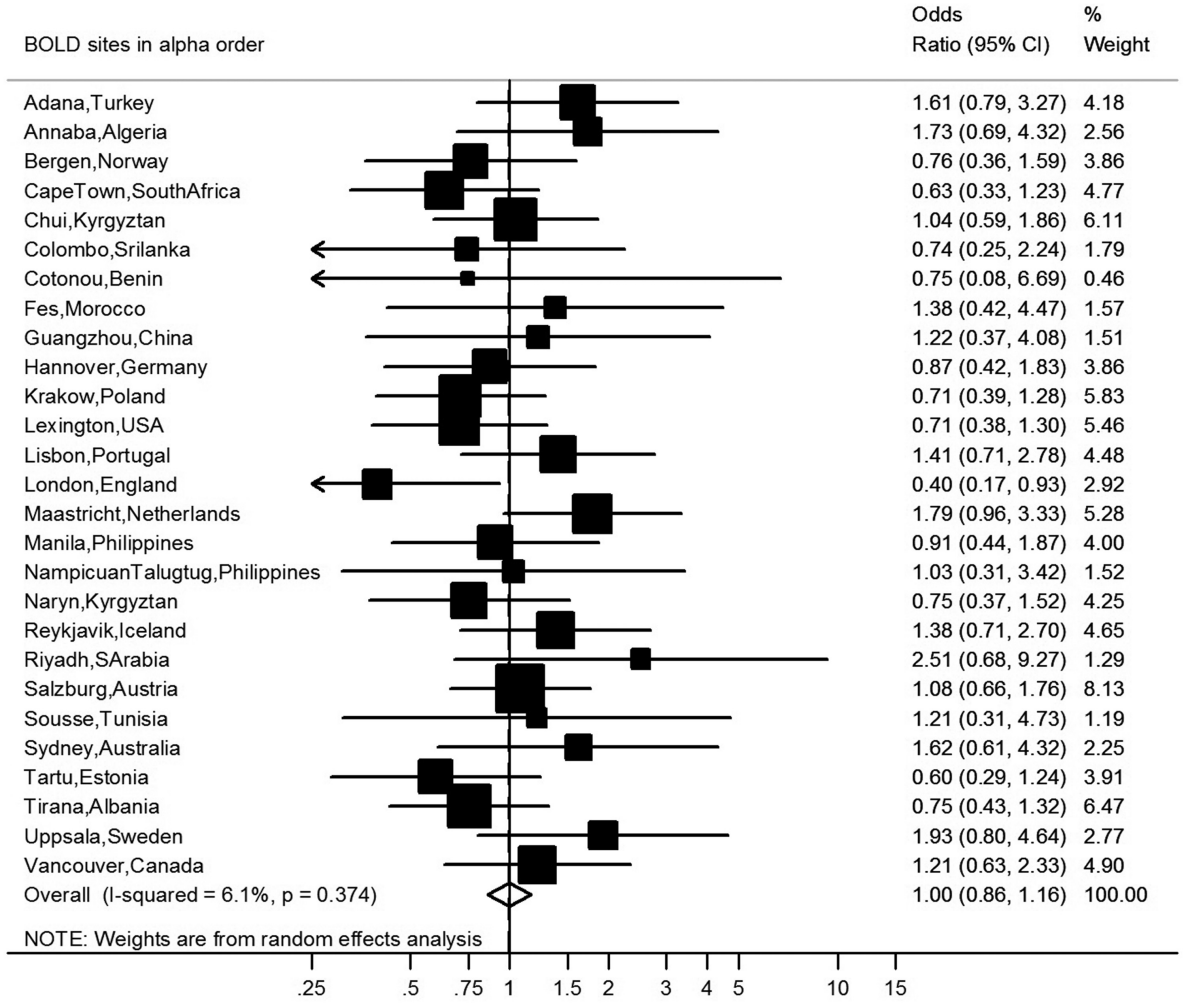
Meta-analysis of the adjusted odds ratios for CVD in subjects with airflow obstruction. Forest plot showing the meta-analysis of odds ratios for CVD, adjusting for age, smoking (pack-years and current smoking status), BMI, education and sex in subjects with airflow obstruction compared to those without airflow obstruction. Heterogeneity chi-squared = 27.68, d.f. = 26 (*P* = 0.374). *I*-squared (variation in ES attributable to heterogeneity) = 6.1%. Estimate of between-study variance Tau-squared = 0.0095. Test for overall effect: *Z* = 0.00 (*P* = 0.999). The following sites could not be included in the analysis due to a low number of subjects reporting CVD or singularity in the data: Blantyre (Malawi), Ife (Nigeria), Mumbai (India), Penang (Malaysia), Pune (India), Srinagar (India).

**Figure 3.**
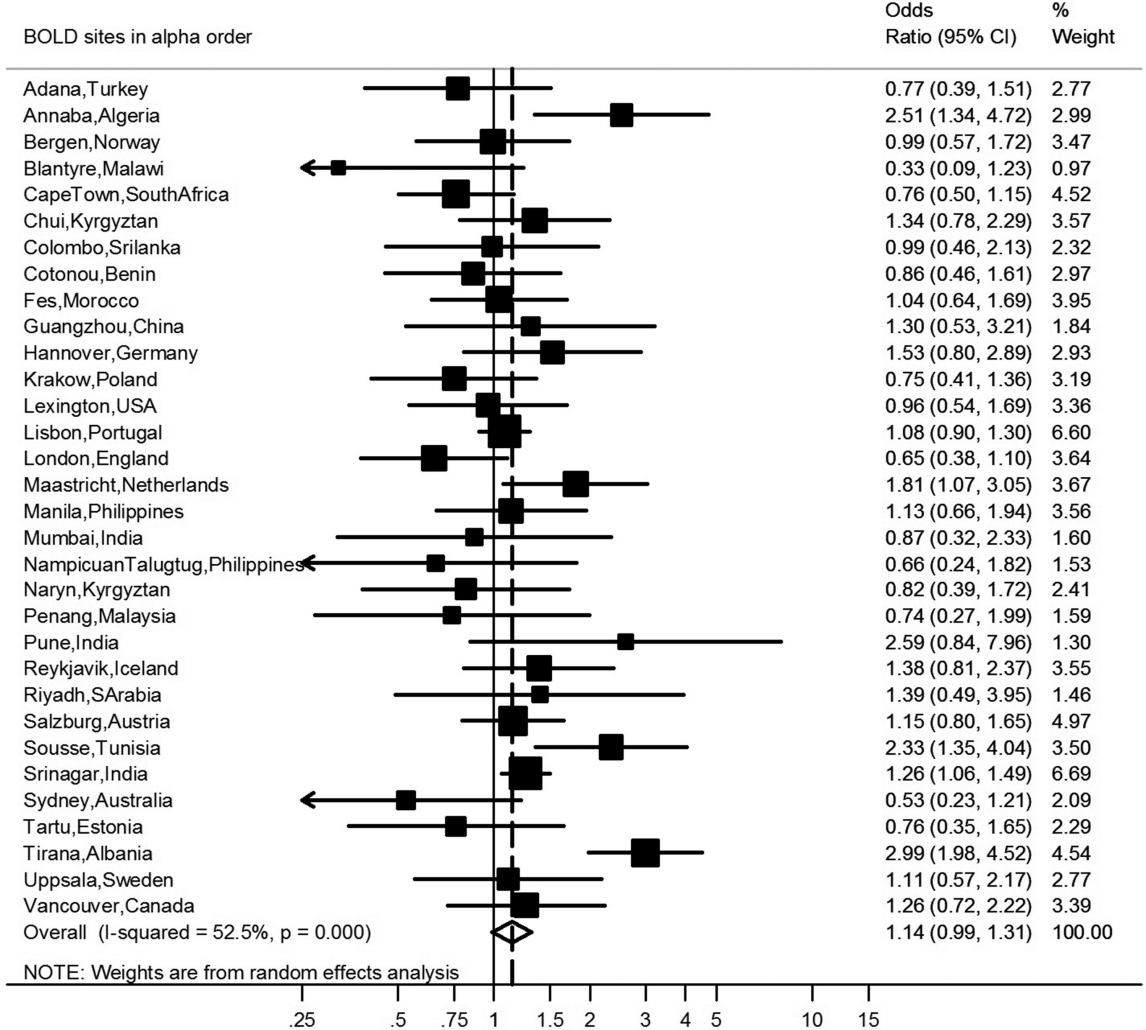
Meta-analysis of the adjusted odds ratios for hypertension in subjects with airflow obstruction. Forest plot showing the meta-analysis of odds ratios for hypertension, adjusting for age, smoking (pack-years and current smoking status), BMI, education and sex in subjects with airflow obstruction compared to those without airflow obstruction. Heterogeneity chi-squared = 65.31, d.f. = 31 (*P* = 0.000). I-squared (variation in ES attributable to heterogeneity) = 52.5%. Estimate of between-study variance Tau-squared = 0.0694. Test for overall effect: *Z* = 1.79 (*P* = 0.074). The following sites could not be included in the analysis due to a low number of subjects reporting hypertension: Ife (Nigeria).

**Figure 4.**
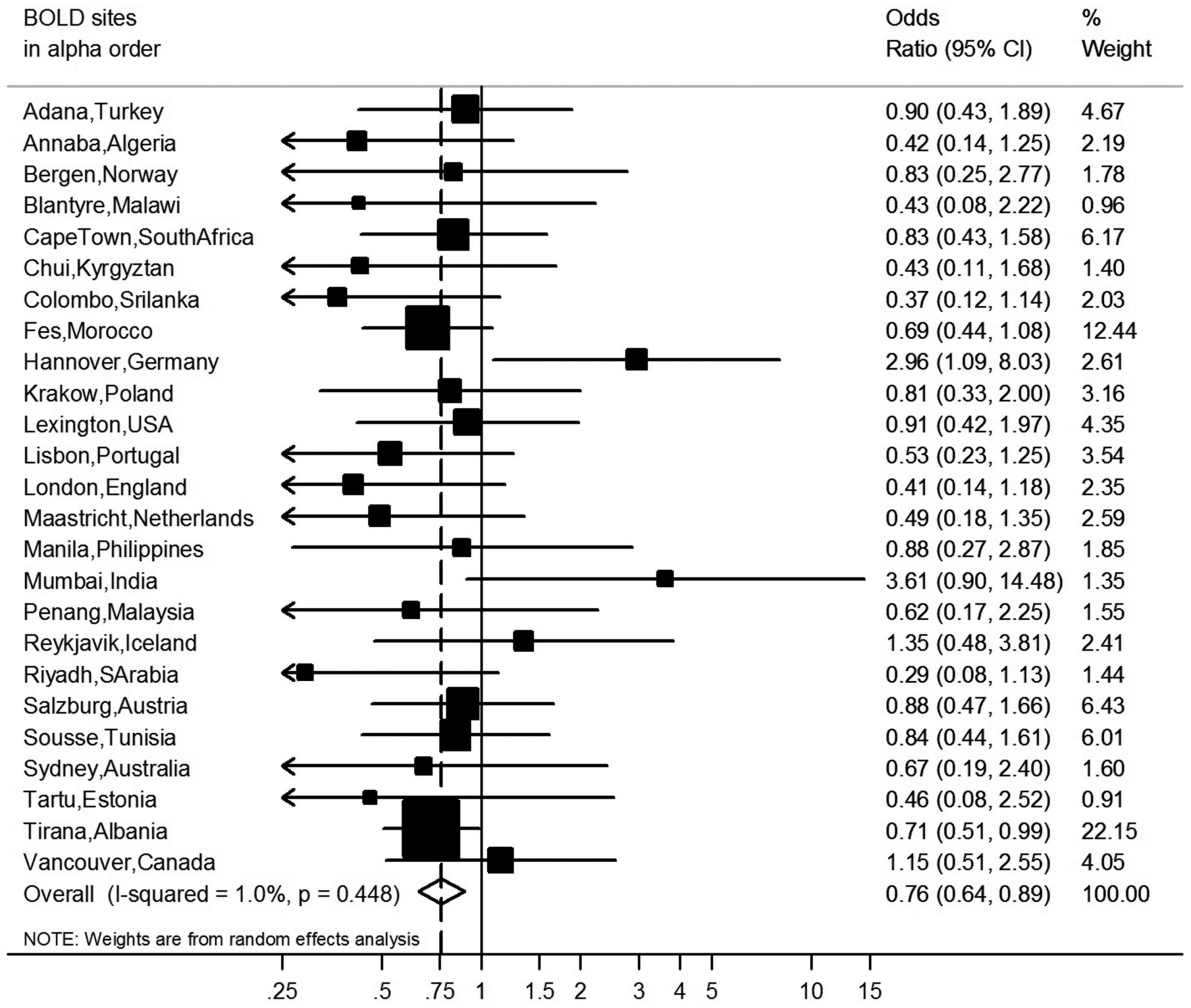
Meta-analysis of the adjusted odds ratios for diabetes in subjects with airflow obstruction. Forest plot showing the meta-analysis of odds ratios for diabetes, adjusting for age, smoking (pack-years and current smoking status), BMI, education and sex in subjects with airflow obstruction compared to those without airflow obstruction. Heterogeneity chi-squared = 24.24, d.f. = 24 (*P* = 0.448). *I*-squared (variation in ES attributable to heterogeneity) = 1.0%. Estimate of between-study variance Tau-squared = 0.0017. Test for overall effect: *Z* = 3.35 (*P* = 0.001). The following sites could not be included in the analysis due to a low number of subjects reporting diabetes or singularity in the data: Cotonou (Benin), Guangzhou (China), Ife (Nigeria), NampicuanTalugtug (Philippines), Naryn (Kyrgyztan), Pune(India), Srinagar (India), Uppsala (Sweden).

**Figure 5.**
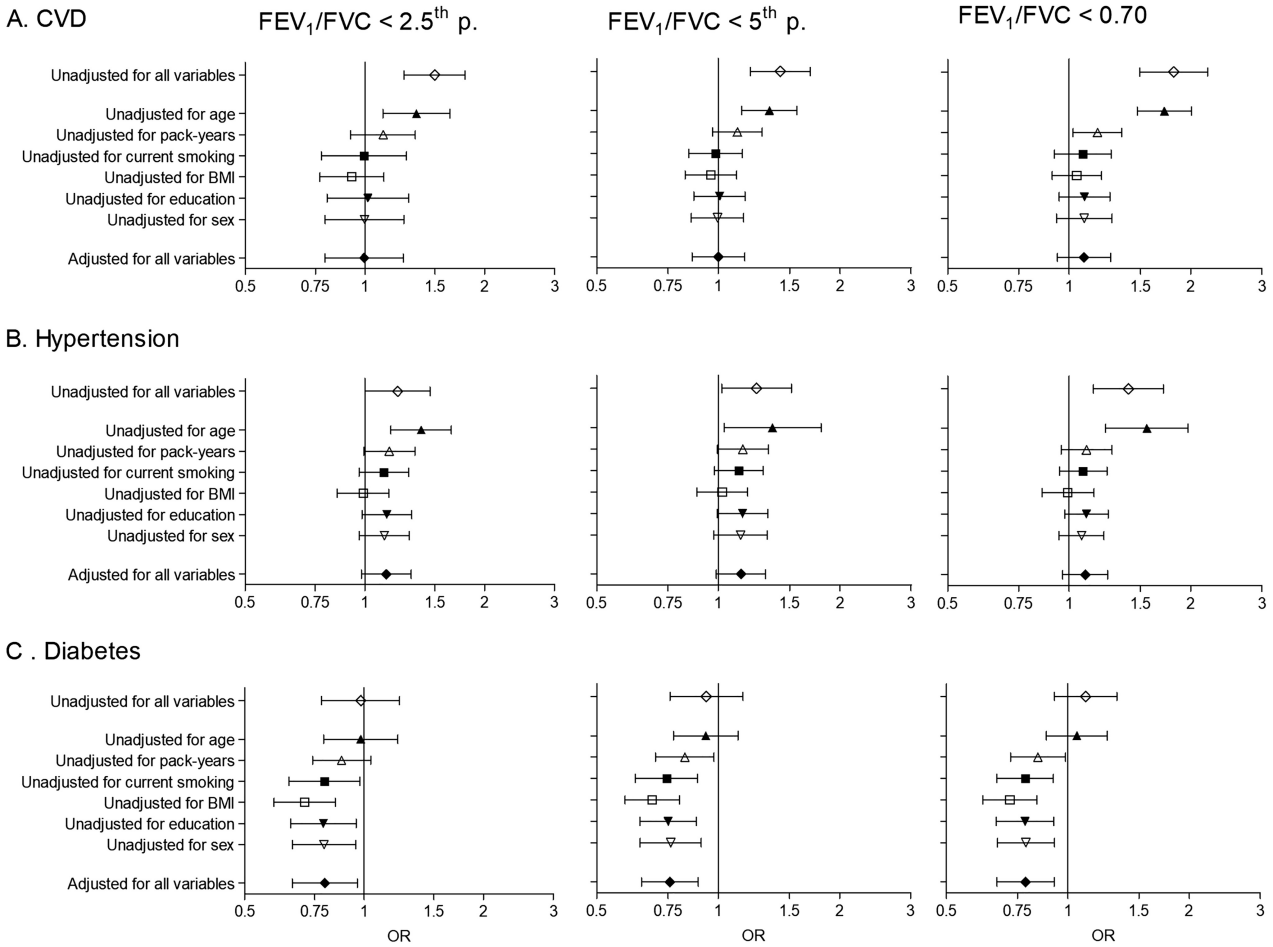
Meta-analyses of the odds ratios for comorbidities in subjects with airflow obstruction, showing the influence of the threshold to define airflow obstruction and adjusting for covariates. Summary forest plots showing the unadjusted, partly adjusted, and completely adjusted odds ratios (meta-OR) and the 95% CI for A. diabetes, B. hypertension, and C. CVD in subjects with airflow obstruction compared to those without airflow obstruction (left column: FEV_1_/FVC < 2.5th percentile, middle column: FEV_1_/FVC < 5th percentile, right column: FEV_1_/FVC < 0.70). Adjusting for all variables at the same time (as shown bottom of each panel), leaving out one variable for each model (middle of each panel), and running one model without any adjusting variables (as shown in the upper part of each panel). The variables age, smoking (pack-years and current smoking status), BMI, education and sex were analyzed. Details of these meta-analyses are reported in Supplementary Table 4.

After covariate adjustment, airflow obstruction was no longer significantly associated with CVD (OR 1.00, 95% CI 0.86–1.16, *I*^2^ = 6.1%, p(heterogeneity) = 0.374) (Figure 2) or hypertension (OR 1.14, 95% CI 0.99–1.31, *I*^2^ = 52%, p(heterogeneity) < 0.001) (Figure 3), but was significantly negatively associated with diabetes (OR 0.76, 95% CI 0.64–0.89, *I*^2^ = 1.0%, p(heterogeneity) = 0.448) (Figure 4). Similar results were found using the alternative definitions for airflow obstruction, although using the fixed ratio (FEV_1_/FVC < 0.70) causes a slight non-significant increase of the risk for CVD (Figure 5).

Age was clearly the most influential of the adjustment variables, followed by pack-years (Figure 5). Adjustment for BMI increased the risk for comorbidities, mainly hypertension and diabetes, in the presence of airflow obstruction (Figure 5). The meta-analyses stratified by sex, smoking and economy showed no significant differences between strata for the odds ratios of airflow obstruction with any of the three comorbidities (Table 2).

**Table 2.** Association of airflow obstruction with comorbidities stratified by sex, smoking history and gross national income.

	Cardiovascular Disease		Hypertension		Diabetes	
	OR	(95% CI)	OR	(95% CI)	OR	(95% CI)
Female	1.08	(0.80–1.46)	1.13	(0.93–1.37)	0.82	(0.61–1.09)
Male	0.97	(0.79–1.20)	1.18	(1.01–1.38)	0.68	(0.49–0.93)
P*	0.576		0.741		0.381	
Ever-smokers	0.98	(0.81–1.19)	1.12	(0.92–1.37)	0.77	(0.58–1.03)
Never-smokers	1.16	(0.89–1.53)	1.09	(0.92–1.29)	0.86	(0.59–1.27)
P*	0.317		0.824		0.651	
Lower income	0.99	(0.79–1.24)	1.20	(0.96–1.50)	0.71	(0.58–0.87)
High income	1.01	(0.79–1.27)	1.06	(0.91–1.24)	0.852	(0.64–1.13)
P*	0.913		0.379		0.304	

* *P* = 2-sided probability of a difference between strata. Lower income: low- & middle- income economies. Details of these meta-analyses are reported in Supplementary Table 2 and 3.

## Discussion

### Main findings

In this international study, we assessed the association between spirometrically identified post-bronchodilator airflow obstruction and the prevalence of diabetes, hypertension, and CVD in the general population, taking into account the potential impact of age, sex, smoking, BMI and education. We demonstrated that CVD and hypertension were more prevalent in subjects with airflow obstruction, but that this association was confounded by known cardiovascular risk factors, principally aging and smoking. In contrast, subjects with airflow obstruction had a lower risk of diabetes after adjusting for age and smoking history. Alternatively defining airflow obstruction by FEV_1_/FVC < 2.5th percentile or 0.70, did not yield significant other results.

### Comparison with other analyses

There are two major problems in interpreting earlier findings. The first is the common use of the spirometric GOLD stages II–IV to classify COPD (2). Introducing the FEV_1_ into the definition of different sub-groups of COPD confuses measurements of obstruction and restriction (10, 18). The current paper intentionally focused on the FEV_1_/FVC ratio, which is a pure measure of airflow obstruction. The second problem is that many studies have relied on comparisons of patient groups (4, 19), which do not reflect associations in the general population (20). Although for these reasons much of the analysis from past studies is difficult to compare with our own results, there is some support from earlier population based surveys for our findings.

#### Cardiovascular disease

Lange et al.’s analysis of the *Copenhagen City Heart Study* (21) reported that both ischemic heart disease, defined as a history of hospital admission for ischemic heart disease or major ischemic alterations on the electrocardiogram, a history of stroke were more prevalent in those with COPD, compared to those without COPD. However, no significant association between COPD and either ischemic heart disease or stroke was noted after adjusting for sex and the older age of COPD subjects. In a study from northern Sweden Lindberg et al. found no evidence of an association between pure airflow obstruction (GOLD stage I COPD) and heart disease, hypertension, stroke, CVD, or diabetes, but only with claudication (22) a condition that has a strong association with smoking (23). In a middle-aged US cohort (*Atherosclerosis Risk in Communities* (ARIC)) of 14,681 subjects, Johnston et al. found no independent association between CVD (self-reported cerebrovascular or ischemic heart disease or evidence of a myocardial infarction on the electrocardiogram) and pure spirometrically defined airflow obstruction (i.e. GOLD stage I COPD), (adjusted OR 1.0 (0.8–1.3) compared to subjects with normal lung function) (24). Corresponding adjusted hazard ratio’s for incident cardiovascular events over 15 years of follow-up were 0.8 (0.5–1.3), 1.1 (0.9–1.5), 1.1 (0.8–1.4) in never, former, and current smokers respectively. In a study that combined the ARIC data with data from the *Cardiovascular Health Study* (CHS), Mannino et al. reported an association between CVD (self-reported cerebrovascular or ischemic heart disease or heart failure) and GOLD stage I COPD (adjusted OR 1.7 (1.5–1.9)) but found no association with either hypertension (adjusted OR 1.1 (0.9–1.2)) or diabetes (adjusted OR 0.9 (0.8–1.1)) (25). In contrast to these findings Garcia-Rio et al., in a population based study in Spain, reported an increased prevalence of self-reported CVD (defined as heart failure, ischemic heart disease, cerebrovascular disease, or peripheral vascular disease), for subjects with both mild COPD (FEV_1_/FVC < 0.70&LLN; FEV_1_ ≥80%)(crude OR 2.53 (1.33–4.76)) and moderate to severe COPD (FEV_1_/FVC < 0.70&LLN; FEV_1_<80%)(crude OR 2.55 (1.51–4.31)) which was not attenuated by adjusting for age, sex, BMI, and smoking (>10 pack-years) (26). It is unclear how far the results were influenced by the inclusion of peripheral vascular disease in this study (23).

#### Hypertension

Hypertension accounts for an estimated 54% of all strokes and 47% of all ischemic heart disease events globally (27). In line with previous reports (4, 22) we found a small increase in self-reported hypertension in subjects with airflow obstruction, though this was not significant after full adjustment (OR = 1.14, 95% CI 0.99–1.31) and there was significant heterogeneity between sites (*I*^2^=52.5%, *P* < 0.001). Adjustment for BMI increased the risk for hypertension in the presence of airflow obstruction (Figure 5, panel B). This might be attributed to the association between airflow obstruction and a lower BMI, as previously shown in the BOLD sample (28). Lange et al. reported higher systolic blood pressure, more use of antihypertensive medication and a higher proportion of subjects with left ventricular hypertrophy as assessed by echocardiography in GOLD stage I COPD (21), but no significant association with left ventricular hypertrophy after adjusting for sex and age (OR = 0.8 (95% CI 0.5–1.3)).

#### Diabetes

Although diabetes prevalence was comparable between subjects with and without airflow obstruction, the adjusted risk for diabetes was lower in subjects with airflow obstruction if their older age was taken into account. In a previous review a slightly increased risk of diabetes (OR 1.36 (1.21–1.53)) has been reported in COPD (4), though the studies reviewed included hospitalized patients. In the population-based studies of Lange (21) and Lindberg (22), a similar prevalence of self-reported diabetes was found in subjects with and without COPD. Although Garcia-Rio et al. reported a 2.5–3 times higher risk of self-reported diabetes in COPD, there was no excess risk after adjustment for age, sex, BMI, and smoking habit (26). Mannino et al. found no excess risk of diabetes in GOLD stage I COPD (OR = 0.9 (0.8–1.1)).

### Strengths

The strength of this study is its size and the heterogeneity of the sample. In spite of this heterogeneity, the results are very consistent and show very little nonrandom variation between sites with the exception of the association between airflow obstruction and hypertension. The association with diabetes is strongly negative (*P* = 0.001) and shows almost no nonrandom variation from site-to-site. There is no evidence that the results for any of the outcomes are different for men and women, smokers and nonsmokers or those living in rich and poor countries (Table 2). Unlike some of the clinical studies the measurement of airflow obstruction is by carefully monitored spirometry, and the samples are general population samples and not recruited from clinical environments.

### Limitations

The main limitation of the study is that the comorbidities are assessed by questionnaire and depend on the participant having been seen by a medical professional, diagnosed and having remembered the diagnosis. However the rates of under-diagnosis would be expected to be higher on average in low income countries and we could find no evidence that the lack of association was particularly associated with low income countries. Like most of the studies that have reported positive associations, these data are cross-sectional, which raises the possibility of survivor bias (i.e. those with more comorbidities being less likely to survive). Such an effect would have to be implausibly large in this relatively young population to fully explain the lack of association.

We have used a single standard equation for each sex for identifying airflow obstruction, the NHANES equations for Caucasian adults. Equations for the ratio of FEV_1_/FVC are very similar for all ethnic groups and using a single standard makes comparisons simpler to interpret. In addition, as the analyses are all made within center and most centers are ethnically homogeneous; this simplification is unlikely to have any substantive effect on the results. Also, the high consistency across sites of the results for CVD and diabetes contribute to their validity.

### Implications

Interest in the possible association between airflow obstruction and cardio-metabolic comorbidities arises from two separate concerns. Clinically it is important to recognize the association so that adequate care can be planned and provided. In this context it is not relevant that the association may be due to confounding by shared risk factors or by the greater tendency of those with multiple pathologies to report to the medical care system. In understanding the origins of these pathologies however and in designing preventive strategies, the explanation of the association as being due to confounding with common risk factors is important.

## Authorship and contributorship

Contributions to conception and design: FJJT, MS, BL, LEGWV; Data analysis: FJJT, MS, WMV, PJGB, LEGWV; Interpretation of data: All authors; Drafting the article or revising it critically for important intellectual content: All authors; Gave final approval of the version to be published: All authors; Take responsibility for the integrity of the data and accuracy of the data analysis: All authors

## Disclosure Statement

The BOLD Study is currently funded by a grant from The Wellcome Trust (085790/Z/08/Z) which supports the London, UK Co-ordinating Center. The initial BOLD program was funded in part by unrestricted educational grants to the Co-ordinating Center in Portland, USA from Aventis, AstraZeneca, Boehringer-Ingelheim, Chiesi, GlaxoSmithKline, Merck, Novartis, Pfizer, Schering-Plough, Sepracor and University of Kentucky. BOLD wishes to acknowledge the contributions of Georg Harnoncourt of the ndd Corporation, Paul Enright, and Bob Jensen for their assistance with spirometry training and quality control during the study. A full list of funders can be found at: http://www.boldstudy.org/sponsors.html. Dr. Filip J.J. Triest, Dr. Michael Studnicka, Dr. Frits M.E. Franssen, Dr. Bernd Lamprecht, and Dr. Lowie E.G.W. Vanfleteren report no other conflicts of interest. Dr. William M. Vollmer reports unrestricted educational grants to the Center for Health Research received unrestricted educational grants from a variety of pharmaceutical companies while serving as Operations Center for the first 15 BOLD sites. These funds were used for training and to pay staff salaries, during the conduct of the study. These are included in the general statement above. Dr. Peter G.J. Burney reports grants from Wellcome Trust, during the conduct of the study. This is included in the general statement above. Dr. Wouters reports personal fees from board membership (Nycomed, Boehringer), grants (AstraZeneca, GSK), personal fees from payment for lectures (AstraZeneca, GSK, Novartis, Chiesi), outside the submitted work.

## References

[1] WHO Assessing national capacity for the prevention and control of noncommunicable diseases: report of the 2015 global survey. Geneva: World Health Organization, 2016.

[2] Vogelmeier CF, Criner GJ, Martinez FJ, et al. Global Strategy for the Diagnosis, Management, and Prevention of Chronic Obstructive Lung Disease 2017 Report. GOLD Executive Summary. Am J Respir Crit Care Med. 2017;195(5):557–582. doi: 10.1164/rccm.201701-0218PP. 28128970

[3] Franssen FME, Soriano JB, Roche N, et al. Lung Function Abnormalities in Smokers with Ischemic Heart Disease. Am J Respir Crit Care Med. 2016;194(5):568–576. doi: 10.1164/rccm.201512-2480OC. 27442601

[4] Chen W, Thomas J, Sadatsafavi M, et al. Risk of cardiovascular comorbidity in patients with chronic obstructive pulmonary disease: a systematic review and meta-analysis. Lancet Respir Med. 2015;3(8):631–639. doi: 10.1016/S2213-2600(15)00241-6. 26208998

[5] Lamprecht B, Soriano JB, Studnicka M, et al. Determinants of underdiagnosis of copd in national and international surveys. Chest. 2015;148(4):971–985. doi: 10.1378/chest.14-2535. 25950276

[6] Buist AS, Vollmer WM, Sullivan SD, et al. The Burden of Obstructive Lung Disease Initiative (BOLD): rationale and design. COPD. 2005;2(2):277–283. 17136954

[7] Perez-Padilla R, Vazquez-Garcia JC, Marquez MN, et al. The long-term stability of portable spirometers used in a multinational study of the prevalence of chronic obstructive pulmonary disease. Respir Care. 2006;51(10):1167–1171. 17005063

[8] American Thoracic Society Standardization of Spirometry, 1994 Update. Am J Respir Crit Care Med. 1995;152(3):1107–1136. 7663792 10.1164/ajrccm.152.3.7663792

[9] Hankinson JL, Odencrantz JR, Fedan KB Spirometric reference values from a sample of the general U.S. population. Am J Respir Crit Care Med. 1999;159(1):179–187. doi: 10.1164/ajrccm.159.1.9712108. 9872837

[10] Coton S, Vollmer WM, Bateman E, et al. Severity of Airflow Obstruction in Chronic Obstructive Pulmonary Disease (COPD): Proposal for a New Classification. COPD. 2017;5:469–475. doi: 10.1080/15412555.2017.1339681. 28799856

[11] Miller MR, Levy ML Chronic obstructive pulmonary disease: missed diagnosis versus misdiagnosis. BMJ. 2015;351:h3021. 26136356 10.1136/bmj.h3021

[12] World Bank World Development Indicators 2015. Washington, DC: World Bank, 2015.

[13] Jousilahti P, Vartiainen E, Tuomilehto J, et al. Sex, age, cardiovascular risk factors, and coronary heart disease: a prospective follow-up study of 14 786 middle-aged men and women in Finland. Circulation. 1999;99(9):1165–1172. 10069784 10.1161/01.cir.99.9.1165

[14] Falkstedt D, Hemmingsson T Educational level and coronary heart disease: a study of potential confounding from factors in childhood and adolescence based on the Swedish 1969 conscription cohort. Ann Epidemiol. 2011;21(5):336–342. doi: 10.1016/j.annepidem.2010.12.005. 21458726

[15] Bogers RP, Bemelmans WE, Hoogenveen RT, et al. Association of overweight with increased risk of coronary heart disease partly independent of blood pressure and cholesterol levels: A meta-analysis of 21 cohort studies including more than 300 000 persons. Arch Intern Med. 2007;167(16):1720–1728. doi: 10.1001/archinte.167.16.1720. 17846390

[16] Tolstrup JS, Hvidtfeldt UA, Flachs EM, et al. Smoking and risk of coronary heart disease in younger, middle-aged, and older adults. Am J Public Health. 2014;104(1):96–102. doi: 10.2105/AJPH.2012.301091. 23763425 PMC3910023

[17] Lubin JH, Couper D, Lutsey PL, et al. Risk of cardiovascular disease from cumulative cigarette use and the impact of smoking intensity. Epidemiology. 2016;27(3):395–404. doi: 10.1097/EDE.0000000000000437. 26745609 PMC5482174

[18] Burney P Coming off the GOLD standard. Lancet Respir Med. 2014;2(3):174–6. doi: 10.1016/S2213-2600(14)70040-2. 24621678

[19] Baty F, Putora PM, Isenring B, et al. Comorbidities and burden of COPD: a population based case-control study. PLoS One. 2013;8(5):e63285. doi: 10.1371/journal.pone.0063285. 23691009 PMC3656944

[20] Berkson J Limitations of the application of fourfold table analysis to hospital data. Biometrics. 1946;2(3):47–53. 21001024

[21] Lange P, Mogelvang R, Marott JL, et al. Cardiovascular morbidity in COPD: a study of the general population. COPD. 2010;7(1):5–10. doi: 10.3109/15412550903499506. 20214458

[22] Lindberg A, Larsson LG, Ronmark E, et al. Co-morbidity in mild-to-moderate COPD: comparison to normal and restrictive lung function. COPD. 2011;8(6):421–428. doi: 10.3109/15412555.2011.629858. 22149402

[23] Joosten MM, Pai JK, Bertoia ML, et al. Associations between conventional cardiovascular risk factors and risk of peripheral artery disease in men. JAMA. 2012;308(16):1660–1667. doi: 10.1001/jama.2012.13415. 23093164 PMC3733106

[24] Johnston AK, Mannino DM, Hagan GW, et al. Relationship between lung function impairment and incidence or recurrence of cardiovascular events in a middle-aged cohort. Thorax. 2008;63(7):599–605. doi: 10.1136/thx.2007.088112. 18245145

[25] Mannino DM, Thorn D, Swensen A, Holguin F Prevalence and outcomes of diabetes, hypertension and cardiovascular disease in COPD. Eur Respir J. 2008;32(4):962–969. doi: 10.1183/09031936.00012408. 18579551

[26] Garcia-Rio F, Soriano JB, Miravitlles M, et al. Overdiagnosing subjects with COPD using the 0.7 fixed ratio: correlation with a poor health-related quality of life. Chest. 2011;139(5):1072–1080. doi: 10.1378/chest.10-1721. 21183609

[27] Lawes CM, Vander Hoorn S, Rodgers A Global burden of blood-pressure-related disease, 2001. Lancet. 2008;371(9623):1513–1518. doi: 10.1016/S0140-6736(08)60655-8. 18456100

[28] Vanfleteren LE, Lamprecht B, Studnicka M, et al. Body mass index and chronic airflow limitation in a worldwide population-based study. Chron Respir Dis. Chron Respir Dis. 2016;13(2):90–101. doi: 10.1177/1479972315626012. 26768010 PMC5734599

